# Improving plant adaptation to soil antimony contamination: the synergistic contribution of arbuscular mycorrhizal fungus and olive mill waste

**DOI:** 10.1186/s12870-024-05044-1

**Published:** 2024-05-04

**Authors:** Mha Albqmi, Samy Selim, Nahla Alsayd Bouqellah, Taghreed S. Alnusaire, Mohammed S. Almuhayawi, Soad K. Al Jaouni, Shaimaa Hussein, Mona Warrad, Mohammad M. Al-Sanea, Mohamed A. Abdelgawad, Ehab M. Mostafa, Mohammad Aldilami, Enas S. Ahmed, Hamada AbdElgawad

**Affiliations:** 1 Department of Chemistry, College of Science, Jouf University, Sakaka, 72341 Saudi Arabia; 2 Olive Research Center, Jouf University, Sakaka, Saudi Arabia; 3 Department of Clinical Laboratory Sciences, College of Applied Medical Sciences, Jouf University, Sakaka, 72341 Saudi Arabia; 4 Science College, Biology Department, Taibah University, Almadina, Almunawwarah 42317-8599 Saudi Arabia; 5 Department of Biology, College of Science, Jouf University, Sakaka, 72341 Saudi Arabia; 6 Department of Clinical Microbiology and Immunology, Faculty of Medicine, King Abdulaziz University, Jeddah, Saudi Arabia; 7 Department of Hematology/Oncology, Yousef Abdulatif Jameel Scientific Chair of Prophetic Medicine Application, Faculty of Medicine, King Abdulaziz University, Jeddah, Saudi Arabia; 8 Department of Pharmacology, College of Pharmacy, Jouf University, Sakaka, Saudi Arabia; 9 Department of Clinical Laboratory Sciences, College of Applied Medical Sciences, Jouf University, Al Qurayyat, Saudi Arabia; 10 Department of Pharmaceutical Chemistry, College of Pharmacy, Jouf University, 72341 Sakaka, Saudi Arabia; 11 Department of Pharmacognosy, College of Pharmacy, Jouf University, 72341 Sakaka, Saudi Arabia; 12 Biology Department, College of Sciences, Majmaah University, 11952 Zulfi, Saudi Arabia; 13 Botany and Microbiology Department, Faculty of Sciences, Beni Suef University, Beni Suef, Egypt

**Keywords:** Antioxidant, Arbuscular mycorrhizal fungus, Chalcone synthase, Oat plant, Olive mill waste, Phenylalanine ammonia-lyase

## Abstract

**Background:**

This study aimed to investigate the alterations in biochemical and physiological responses of oat plants exposed to antimony (Sb) contamination in soil. Specifically, we evaluated the effectiveness of an arbuscular mycorrhizal fungus (AMF) and olive mill waste (OMW) in mitigating the effects of Sb contamination. The soil was treated with a commercial strain of AMF (*Rhizophagus irregularis*) and OMW (4% w/w) under two different levels of Sb (0 and 1500 mg kg^−1^ soil).

**Results:**

The combined treatment (OMW + AMF) enhanced the photosynthetic rate (+ 40%) and chlorophyll a (+ 91%) and chlorophyll b (+ 50%) content under Sb condition, which in turn induced more biomass production (+ 67–78%) compared to the contaminated control plants. More photosynthesis in OMW + AMF-treated plants gives a route for phenylalanine amino acid synthesis (+ 69%), which is used as a precursor for the biosynthesis of secondary metabolites, including flavonoids (+ 110%), polyphenols (+ 26%), and anthocyanins (+ 63%) compared to control plants. More activation of phenylalanine ammonia-lyase (+ 38%) and chalcone synthase (+ 26%) enzymes in OMW + AMF-treated plants under Sb stress indicated the activation of phenylpropanoid pathways in antioxidant metabolites biosynthesis. There was also improved shifting of antioxidant enzyme activities in the ASC/GSH and catalytic pathways in plants in response to OMW + AMF and Sb contamination, remarkably reducing oxidative damage markers.

**Conclusions:**

While individual applications of OMW and AMF also demonstrated some degree of plant tolerance induction, the combined presence of AMF with OMW supplementation significantly enhanced plant biomass production and adaptability to oxidative stress induced by soil Sb contamination.

## Introduction

Antimony (Sb) is a naturally occurring trace metalloid in the environment, where its concentration in soils, sedimentary rocks, and aquatic environments ranges about 0.3–8.6 mg kg^−1^, 0.15–2 mg kg^−1^, and 1 µg mL^−1^, respectively [1, 2]. More recent attention has focused on environmental contamination by increasing concentration of Sb due to its extensive release from various industrial processes, leaching of mining wastes, weathering of sulfide ores, and anthropogenic activities [2, 3], which resulted in accumulation of Sb to 5000 mg kg^−1^ in some polluted soils [4]. Since two oxidation forms of Sb, including Sb(III) and Sb(V), can be absorbed by crop plants from the soil, it can be potentially dangerous to public health via the accumulation in the food chain [5]. Besides that, high levels of Sb in the soil can exert a detrimental impact on crop growth and productivity, because its absorption, transportation, and sequestration by plants can affect all aspects of plant life [5].

Sb in plants has no beneficial physiological role and can also cause many adverse physiological effects on plants such as impairing the function of the electron transport chain, disturbing redox equilibrium, and eventually resulting in the accumulation of reactive oxygen species (ROS) [6], which can consequently cause a disturbance in photosynthesis pathway, and decline in plant growth and biomass production [7]. Plants employ various mechanisms to cope with heavy metal stress, such as enzymatic and non-enzymatic antioxidant defense systems [8, 9]. This led to accumulating protective metal-binding metabolites, metal chelates in vacuoles or emitting them into the rhizosphere [8]. Also allocating the primary less functional carbohydrate and amino acids to structural carbohydrates and bioactive secondary metabolites [10].

Soil-beneficial microbes are of interest for utilization in sustainable agriculture as biofertilizers or biopesticides as well as phytoremediater [4, 11, 12]. Among them, arbuscular mycorrhizal fungi (AMF) have been proposed as stress-tolerant bioactive microorganisms since they not only enhance plant growth mainly through improving nutrient uptake from the soil but also alleviate the damaging impacts of stressors by promoting plant resistance [13, 14]. Although the molecular basis of to Sb stress mitigating mechanisms by AMF is unclear yet, the ability of AMF in remediation of soil heavy metals, reduction of metal toxicity incurred by host plants, and immobilize or mobilize heavy metals in the soil has been already documented [4, 14, 15]. In this regard, it has been reported that most plants grown near the Sb mining sites have formed symbiotic associations with AMF [16]. This is a positive correlation was found between Sb concentrations in plants and the degree of AMF colonization [16, 17].

Moreover, the emphasis on agricultural waste recovery and recycling has been one of the main debates in sustainable agriculture [9]. The topic of the organic amendments production for soil application from olive waste, containing an optimal level of nutritional values, received considerable critical attention within the scientific society [18]. One of those agricultural and food waste is olive waste which is generated by the olive oil manufacturing process and is known for its favourable impacts on plants, mainly under soil heavy metal contamination [9, 19].

In this context, olive waste has recently been employed as a soil supplementation treatment, not only to enhance soil organic matter content [9, 20] but also due to its antimicrobial and antiviral properties [21].

Despite the earlier findings outlined above, little is known about Sb contamination and it is not clear to what extent the synergic effects of olive waste and beneficial arbuscular mycorrhizal fungus in soils can relieve its toxic impacts on plants through the modification of metabolites and biochemical composition of plants. The objectives of this study are twofold: (i) to investigate whether the oxidative stress induced by Sb toxicity in oat (*Avena sativa*) plants can be mitigated through treatments involving a commercial AMF and/or OM, (ii) to determine whether the alteration of plant metabolic parameters and biochemical compounds is more pronounced in oat plants subjected to the combined application of AMF and OMW compared to their individual applications. We hypothesized that the combined application of AMF and OMW will enhance oat plant responses to Sb-contaminated soil, leading to improved photosynthetic rate, biomass production, and synthesis of secondary metabolites compared to plants treated with either AMF or OMW alone or left untreated. By investigating the biochemical and physiological responses of oat plants to Sb contamination, the present research tries to offer valuable insights into sustainable agricultural practices aimed at addressing soil contamination issues and improving crop productivity in contaminated areas.

## Material and methods

### Plant materials and experimental setup

A pure commercial inoculum of arbuscular mycorrhizal fungus (AMF) strain, *Rhizophagus irregularis* (MUCL 41833), was provided from Glomeromycota in vitro Collection (GINCO) (www.mycorrhiza.be/ginco-bel) in a pot (with a diameter and height of 25 cm). The AMF inoculum was added as 10 g of trapped soil per pot (approx. 50 spores g^−1^ soil). Healthy uniform seeds of oat (*Avena sativa* L. var sids 107), which were obtained from the Agricultural Research Center (Giza, Egypt), were planted in pots containing sterilized sand soil (70%) and Tref EGO substrates (Moerdijk, Netherland; 30%). The inoculation treatment was divided into two levels including (i) soil treatment with AMF inoculants and (ii) control treatment by adding the autoclaved inoculum in equal amounts to provide equivalent nutrients aside from mycorrhizal spores. To apply OMW treatment, soils were supplemented with 4% w/w of OMW after collecting from a traditional and air-drying for one month before use [19]. The physicochemical properties of OMW are presented in Table 1.

**Table 1:** Basic physicochemical properties of olive mill waste (± standard deviation)

*Characteristics*	
Dry matter (%)	18.73 ± 2.16
pH	6.97 ± 0.39
Electric conductivity (mS cm^−1^)	16.08 ± 1.66
Chemical oxygen demand (g L^−1^)	103.18 ± 8.47
Biochemical oxygen demand (g L^−1^)	61.97 ± 5.02
Organic matter (g L^−1^)	51.91 ± 5.88
*Minerals*	
Nitrogen (g L^−1^)	4.86 ± 0.56
Phosphorus (g L^−1^)	6.30 ± 0.66
Potassium (g L^−1^)	9.96 ± 0.83
Calcium (g L^−1^)	2.98 ± 0.62
Magnesium (g L^−1^)	2.34 ± 0.31
Fe (g L^−1^)	1.10 ± 0.18
Zn (g L^−1^)	0.88 0.09
*Antioxidants*	
Antioxidant activity (FRAP)	64.38 ± 5.11
Antioxidant activity (DPPH)	82.95 ± 7.88
Total phenols (g L^−1^)	17.56 ± 2.77
Total flavonoids (g L^−1^)	1.92 ± 0.08

Sb was also added to the soil as Sb(V) (potassium hexahydroxoantimonate (V); KSb (OH)_6_) at a concentration of 1500 mg kg^−1^ of soil, based on the preliminary studies on the effect of a wide range of Sb concentrations (100–1500 mg kg^−1^) on cereal growth by inducing a progressively adverse impact with > 50% dead plants after two weeks [5]. The three pots represented three biological replicates for each treatment. All the pots were weighed daily to keep the water level (> 60% of field capacity) throughout the experiment. The plant growth conditions were set in a growth chamber with a regime of 21/18 °C in a 16/8 h day/night photoperiod and 350 μmol PAR m^−2^ s^−1^ [22]. The fresh weight (FW) and dry weight (DW) of shoots were collected after four weeks of growth, and kept at -80 °C for further examinations.

## Mycorrhizal colonization

The percentage of mycorrhizal colonization in plant roots was determined following the method described by Giovannetti and Mosse (1980). Root samples were cut into 1 cm bits and fixed in FAA solution (Formalin: Acetic acid: Alcohol; 5:5:90 v/v/v). The roots were then cleared by autoclaving in 10% KOH solution for 15 min, followed by neutralization with 1% HCl for 5 min. Staining was performed by simmering the roots in 0.05% trypan blue in lactoglycerol for 10 min. Stained root bits were mounted on slides and examined under a microscope to observe hyphal and arbuscules. Ten randomly selected stained root pieces of 1 cm length each were arranged on a slide. The length of fungal infection was assessed in centimeters for each root piece, averaged across ten pieces, and expressed as a percentage of colonization [23].

## Determination of photosynthetic related parameters in wheat plants

Some photosynthetic traits were assessed to investigate the impacts of beneficial actinobacteria and heat stress on plant biomass production. In this regard, the light-saturated photosynthetic rate of the most youthful expanded leaves was analyzed by a LI-COR portable photosynthesis system (LI-COR 6400/XT, USA). The concentration of chlorophyll *a* (Chl *a*) and chlorophyll *b* (Chl *b*) pigments were measured at 665 and 652 nm, respectively [24].

## Determination of oxidative stress markers

To estimate malondialdehyde (MDA) concentration in the shoot tissues, the fresh samples were homogenized in ethanol (80% v/v) at 7000 g for 60 s using a MagNALyser (Roche, Vilvoorde, Belgium) and reacted with thiobarbituric acid (TBA) to form pinkish red chromogen thiobarbituric acid‒malondialdehyde. The absorbance of the final product was measured at 440, 532, and 600 nm [25]. The content of hydrogen peroxide (H_2_O_2_) in the shoots was also measured in trichloroacetic acid (0.1% v/v) on the basis of peroxide-catalyzed oxidation of Fe^2+^, according to the xylenol orange method [26].

## Determination of the overall antioxidants

Total antioxidant capacity in the shoot samples was measured using ferric reducing/antioxidant power (FRAP) reagent, containing 0.3 M acetate buffer (pH 3.6), 0.01 mM 2,4,6‒Tris(2‒pyridyl)‒s‒triazine (TPTZ) in 0.04 mM HCl and 0.02 M FeCl3, followed by reading the absorbance of extracted samples at 600 nm [27]. The standard was 6‒hydroxy‒2,5,7,8‒tetramethylchromane‒2‒carboxylic acid (Trolox). Also, gallic acid and quercetin standards were used to measure the content of total polyphenols and flavonoids in the shoot samples, as described by Zhang et al. [28] and Chang et al. [29], respectively. Dimethyl tocol was considered an internal standard to measure total tocopherols by HPLC (SCL-10 AVP, Shimadzu Corporation, Kyoto, Japan) [30]. HPLC method was employed to estimate reduced glutathione (GSH) and reduced ascorbate (ASC) [31, 32]. The activities of antioxidant enzymes were assessed in a semi-high-throughput set-up [26, 33]. The activity of superoxide dismutase (SOD) was measured by estimating the inhibition of nitro-blue tetrazolium (NBT) reduction (ɛ550 = 12.8 mM^–1^ cm^–1^) [34]. The oxidation of pyrogallol in phosphate buffer at 430 nm was analyzed to measure the activity of peroxidase (POX); ɛ430 = 2.46 mM^–1^ cm^–1^) [34]. The activity of catalase (CAT) was assessed by observing the H_2_O_2_ decomposition at 240 nm (ɛ240 = 39.4 M^−1^ cm^−1^) [35]. The estimation of ascorbate peroxidase (APX) activity was done by recording the decrease in absorbance at 290 nm (ɛ290 = 2.8 mM ^−1^ cm ^−1^) as fully described by Nakano and Asada et al. [36]. The decrease in NADPH at 340 nm was monitored to estimate the activity of glutathione reductase (GR; ɛ340 = 6.22 mM^–1^ cm^–1^) [37]. The reduction in NADPH absorption was evaluated to measure the activity of glutathione peroxidase (GPX), in a coupled enzyme assay with GR, as described by Drotar et al. [38].

## Determination of anthocyanins, phenolics and flavonoids

To measure the anthocyanins content, methanol: HCl (99:1 v/v) was used for homogenizing the samples, followed by incubating the extracted samples at 25 °C for 24 h in the dark conditions and centrifuging at 4000 g for 5 min. The absorbance of the extracted supernatant was read at 550 nm (ɛ550 = 233,000 M^−1^ cm^−1^) [39]. Individual flavonoids and phenolic acids were analyzed by HPLC with a Lichrosorb Si-60 column (7 μm, 3 × 150 mm) and DAD detector, as fully explained by Hamad et al. [40]. Briefly, acetone:water (4:1, v/v) solution was used for homogenizing the shoot samples for 24 h. The final extracted samples were subjected to HPLC at 0.8 mL min^−1^ (flow rate), in which the mobile phase consisted of water:formic acid (90:10 v/v) and acetonitrile:water:formic acid (85:10:5 v/v/v). A calibration curve of the related standard was considered to estimate the content of each compound. The activity of phenylalanine amino-layse (PAL) was measured by extracting the samples in sodium borate buffer and reading the absorbance of trans-cinnamic acid output at 290 nm [40].

## Statistical analysis

SPSS statistical package (SPSS Inc., Chicago, IL, USA) was used for the statistical analysis, including the two-way (ANOVA) and Tukey HSD post-hoc test at 5% probability level (*p* < 0.05). The values represent the mean of at least three replicates (n ≥ 3).

## Results

### Plant biomass and photosynthetic parameters

The results of the analysis of the root colonization by AMF are presented in Table 2. In this regard, Sb contamination in soil significantly decreased root colonization (− 78%), hyphal length (− 87%), and number of arbuscules (− 80%) compared to AMF-treated plants under non-stress conditions. Nevertheless, OMW supplementation significantly improved the root colonization and hyphal length in AMF-treated plants, up to 25% and 29%, respectively, compared to those left untreated with OMW (Table 2).

**Table 2:** The effect of arbuscular mycorrhizal fungus (AMF) and olive mill waste (OMW) on root colonization (%), hyphal length (cm g^−1^ soil) and number of arbuscules (no. cm^−1^ root) under non-stress and antimony stress conditions

	Non-stress	Antimony stress	
	AMF	AMF	OMW + AMF
Root colonization	59.35 ± 0.86 a	33.38 ± 1.90 c	41.63 ± 2.49 b
Hyphal length	25.76 ± 2.91 a	13.74 ± 1.08 c	17.71 ± 1.42 b
Number of arbuscules	6.53 ± 0.54 a	3.63 ± 0.39 b	4.19 ± 0.40 b

The means in each parameter with similar small letter(s) are not significantly different at 5% probability level (Tukey's HSD test)

**Fig. 1:**
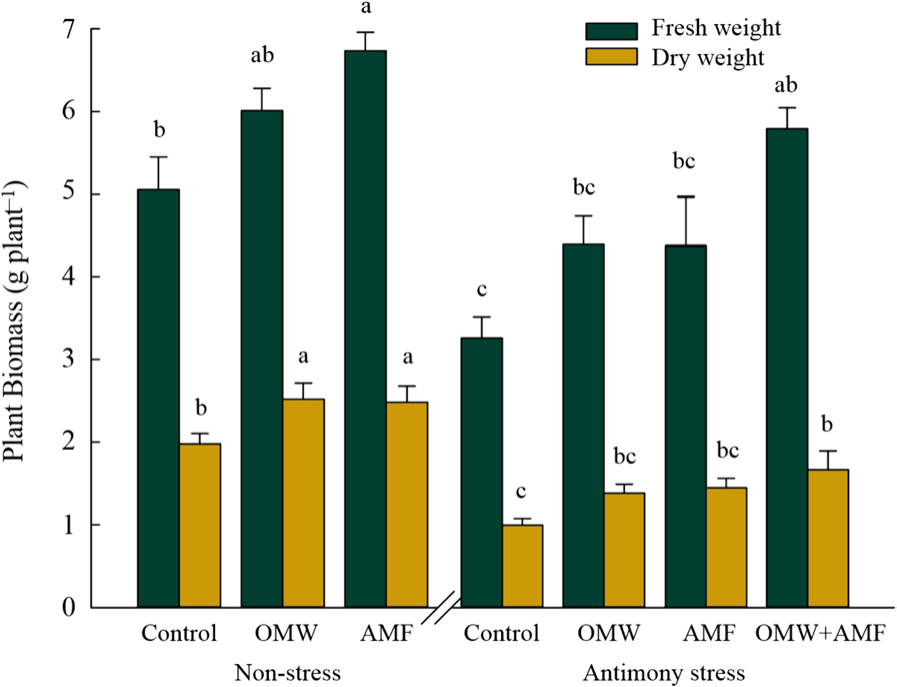
The effect of arbuscular mycorrhizal fungus (AMF) and olive mill waste (OMW) on plant biomass in oat plants. The means in each parameter with similar small letter(s) are not significantly different at 5% probability level (Tukey HSD test). FW: Fresh weight; DW: Dry weight

As can be seen from Fig. 1, Sb contamination resulted in a sharp decline (*p* < 0.05) in the fresh and dry weights of control oat plants, equal to -35% and -50%, respectively. However, AMF treatment in non-contaminated soil significantly (*p* < 0.05) improved FW (+ 33%) and DW (+ 26%) compared to control plants at the same stress level. Its impact in the contaminated soil was not significant ((*p* > 0.05). Moreover, the highest amount of FW and DW were obtained from the combined treatment (OMW + AMF) which were 78% and 67% higher than control plants in the contaminated soil.

**Fig. 2:**
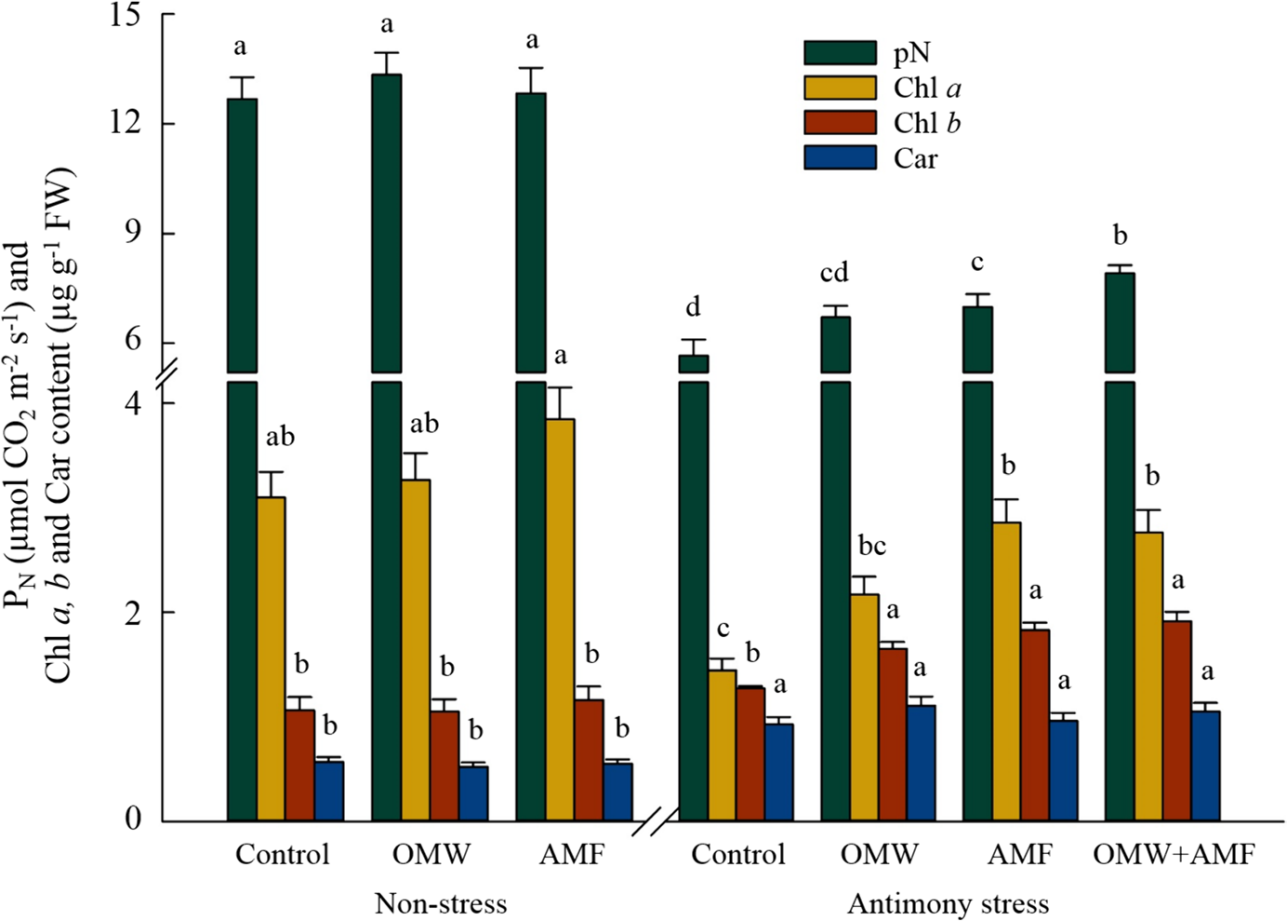
The effect of arbuscular mycorrhizal fungus (AMF) and olive mill waste (OMW) on the photosynthesis parameters, including photosynthesis rate (P_N_) and the content of chlorophyll *a* (Chl *a*)*,* Chlorophyll *b* (Chl *b*)*,* and carotenoids (Car) in oat plants. The means in each parameter with similar small letter(s) are not significantly different at 5% probability level (Tukey HSD test)

Similarly, Fig. 2 revealed that there has been a steep drop in the photosynthesis rate (-55%) and chlorophyll *a* content (-55%) in control plants treated with Sb (*p* < 0.05). However, the application of AMF treatments resulted in higher values of photosynthesis rate, chlorophyll *a* and *b* content under Sb stress conditions (*p* < 0.05), with an improvement of 24%, 98%, and 44% in AMF and 40%, 91% and 50% in OMW + AMF treatments compared to control, respectively (Fig. 2). Although bio-inoculation/amendment treatments have no effects on carotenoids content in leaves (*p* > 0.05), Sb contamination significantly improved its content compared to non-contamination conditions (+ 62–94%; *p* < 0.05).

## Oxidative stress markers and antioxidant systems

**Fig. 3:**
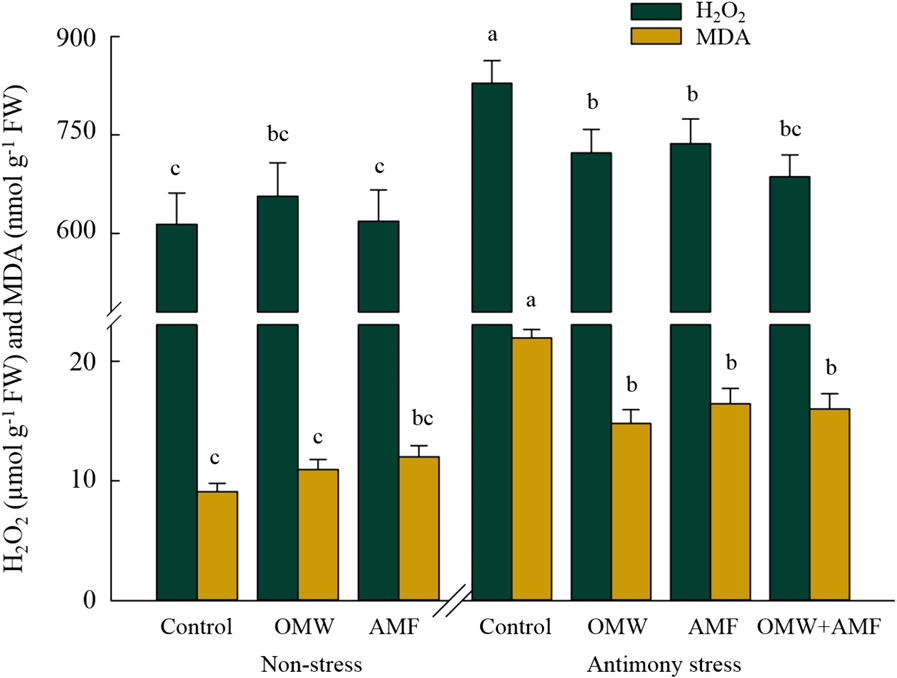
The effect of arbuscular mycorrhizal fungus (AMF) and olive mill waste (OMW) on the oxidative markers, including hydrogen peroxide (H_2_O_2_) and malondialdehyde (MDA) in oat plants. The means in each parameter with similar small letter(s) are not significantly different at 5% probability level (Tukey HSD test)

Figure 3 presents vivid proof of oxidative damage in plants grown under Sb contamination conditions (*p* < 0.05), where the content of H_2_O_2_ and MDA were 35% and 141% higher than those non-contaminated plants, respectively (Fig. 3). The most intriguing discovery is the significant decrease (*p* < 0.05) in H_2_O_2_ and MDA levels observed in plants treated with bio-inoculation/amendment under contaminated conditions, compared to control contaminated-plants, showing reductions of approximately -13% and -32% in OMW, -11% and -25% in AMF, and -17% and -27% in OMW-AMF treatments, respectively.

The activity of antioxidant enzymes, including the direct ROS-detoxifying (POX, SOD, and CAT) and those involved in the ascorbate–glutathione (ASC-GSH) pathway (APX, DHAR, MDHAR, GR, and GPX) was assessed to disclose the plant strategies in avoiding stress caused by Sb stress (Table 3). In this regard, the oxidative damage caused by contamination significantly increased (*p* < 0.05) the activity of CAT (+ 27%), SOD (+ 35%), DHAR (+ 99%), and GR (+ 47%) compared to those grown under control condition. While not affecting (*p* > 0.05) POD (+ 17%), APX (+ 22%), MDHAR (+ 2%), and GPX (+ 4%). Although the individual application of OMW and AMF significantly improved the activity of most studied enzymes, The combined treatment (OMW + AMF) resulted in the highest activity of POX, CAT, SOD, APX, DHAR, MDHAR, GR, and GPX enzymes under stress, exhibiting increases of + 104%, + 179%, + 26%, + 113%, + 53%, + 46%, + 57%, and + 37%, respectively, compared to control stressed plants.

**Table 3:** The effect of arbuscular mycorrhizal fungus (AMF) and olive mill waste (OMW) on the antioxidant direct ROS-scavenging enzymes (represented as μmol min^−1^ mg^−1^ protein for POX and CAT, and mmol min^−1^ mg^−1^ protein for SOD) and the enzymatic components (μmol min^−1^ mg^−1^ protein) of the ascorbate–glutathione (ASC/GSH) and catalytic cycles in oat plants

	Non-stress			Antimony stress					
	Control	OMW	AMF	Control	OMW		AMF		OMW + AMF
POX	2.19 ± 0.14 c	2.15 ± 0.17 c	2.03 ± 0.16 c	2.57 ± 0.20 c	3.51 ± 0.21b		3.87 ± 0.18 b		5.22 ± 0.41a
CAT	12.19 ± 0.95 e	11.71 ± 0.92 e	14.60 ± 0.86 d	15.51 ± 1.12 d	24.00 ± 1.78 c		35.59 ± 1.85 b		43.32 ± 2.38 a
SOD	318.82 ± 24.87 c	321.95 ± 25.01 c	355.78 ± 26.75 c	430.78 ± 33.06 b	423.06 ± 32.11 b		588.56 ± 45.91 a		542.32 ± 52.30 a
APX	0.55 ± 0.03 c	0.52 ± 0.04 c	0.58 ± 0.05 c	0.66 ± 0.05 c	1.35 ± 0.08 a		0.96 ± 0.07 b		1.41 ± 0.11 a
DHAR	0.25 ± 0.02 d	0.23 ± 0.01 d	0.28 ± 0.02 cd	0.39 ± 0.04 c	0.54 ± 0.04 b		0.59 ± 0.04 b		0.75 ± 0.05 a
MDHAR	0.07 ± 0.01 b	0.08 ± 0.01 b	0.09 ± 0.01 ab	0.07 ± 0.01 b	0.13 ± 0.01 a		0.11 ± 0.01 a		0.13 ± 0.01 a
GR	0.12 ± 0.01 c	0.19 ± 0.01b	0.16 ± 0.01 bc	0.18 ± 0.01 b	0.25 ± 0.02 ab		0.22 ± 0.02 ab		0.28 ± 0.02 a
GPX	0.81 ± 0.06 b	0.77 ± 0.06 b	0.79 ± 0.06 b	0.85 ± 0.05 b	1.16 ± 0.09 a		1.19 ± 0.08 a		1.16 ± 0.09 a

The means in each parameter with similar small letter(s) are not significantly different at 5% probability level (Tukey's HSD test)

*POX* Peroxidase, *CAT* Catalase, *SOD* Superoxide dismutases, *GPX* Glutathione peroxidase, *APX* Ascorbate peroxidase, *GR* Glutathione reductase, *DHAR* Dehydroascorbate reductase, *MDHAR* Monodehydroasorbate reductase

**Fig. 4:**
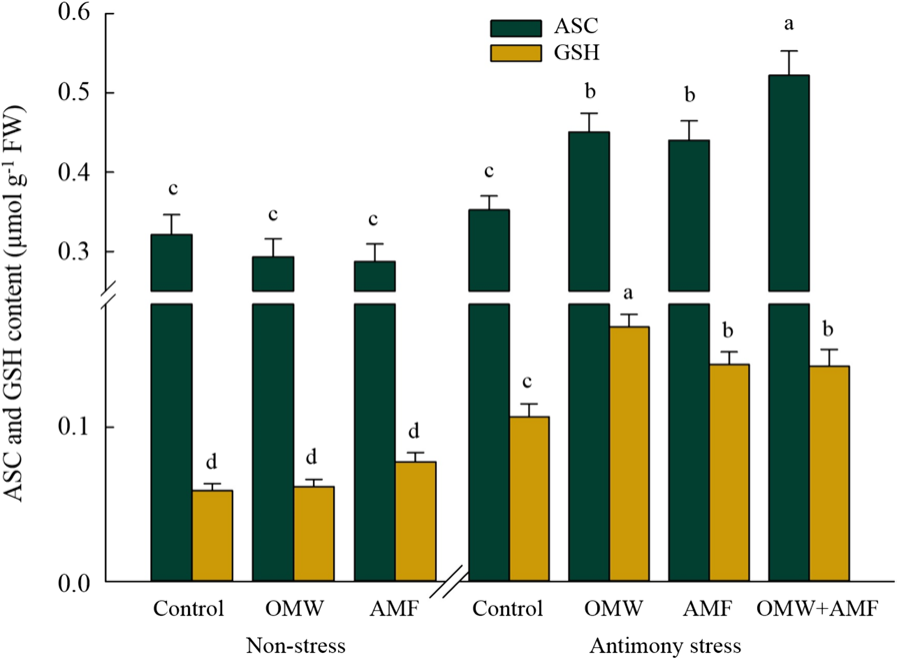
The effect of arbuscular mycorrhizal fungus (AMF) and olive mill waste (OMW) on non-enzymatic component of the ascorbate–glutathione (ASC/GSH) cycle in oat plants. The means in each parameter with similar small letter(s) are not significantly different at 5% probability level (Tukey HSD test). ASC: Ascorbate; GSH: Gluthatione

The content of antioxidant proteins involved in the ASC/GSH cycle, ASC and GSH, reacted differently to the Sb contamination conditions and bio-inoculant/amendment treatments (Fig. 4). Accordingly, the Sb stress caused an increase (*p* < 0.05) in the accumulation of GSH (+ 82%) in plants compared to non-stress conditions, while it did not affect ASC content (+ 10%) (*p* > 0.05). Notwithstanding that, all bio-inoculant/amendment treatments under stress significantly (*p* < 0.05) improved the accumulation of these proteins, the highest content of ASC and GSH belonged to the OMW + AMF and OMW treatments, which were 48% and + 54% higher than control plants grown in contamination conditions, respectively.

**Fig. 5:**
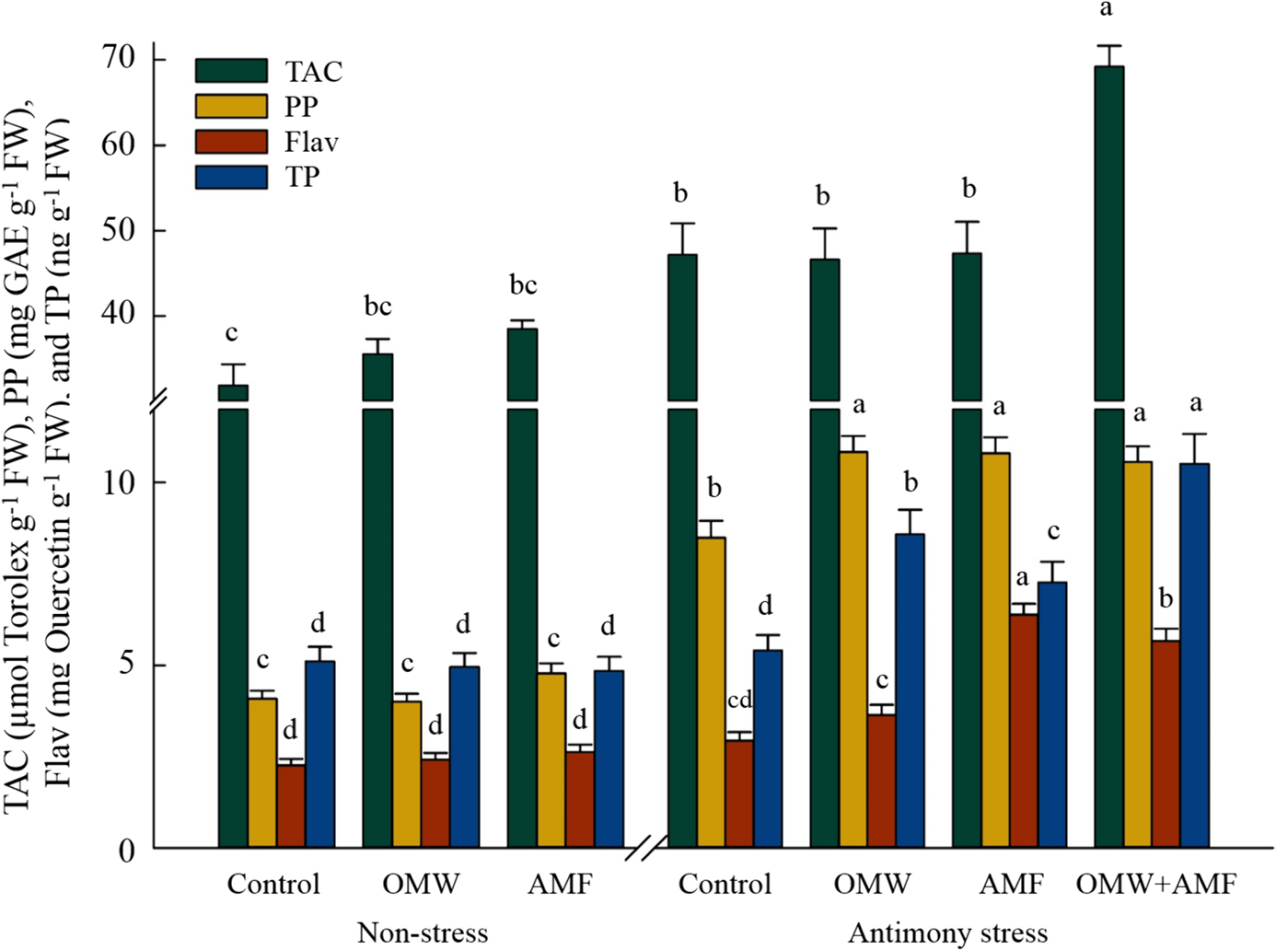
The effect of arbuscular mycorrhizal fungus (AMF) and olive mill waste (OMW) on the antioxidant metabolites in oat plant. The means in each parameter with similar small letter(s) are not significantly different at 5% probability level (Tukey HSD test). TAC: Total antioxidant capacity; PP: Polyphenols; Flav: Flavonoids; TP: Tocopherols

Although Sb contamination did not affect (*p* > 0.05) total flavonoids and tocopherols content, it significantly (*p* < 0.05) increased total antioxidant capacity (+ 48%) and total polyphenols (+ 108%) as compared to non-contaminated plants (Fig. 5). The highest accumulation of TAC and total tocopherols under Sb stress conditions was obtained from OMW + AMF treatment which was significantly (*p* < 0.05) higher than the individual application of OMW (+ 48% and + 22%, respectively), AMF (+ 46% and + 45%, respectively) and control (+ 47% and + 94%, respectively) treatments under stress. Moreover, the content of total polyphenols in stressed plants was considerably (*p* < 0.05) affected by both OMW (+ 27%) and AMF (+ 28%) compared to control plants (Fig. 5). The highest accumulation of flavonoids in the stressed plants belonged to the AMF treatment, which was placed in the same statistical group with OMW-AMF (*p* > 0.05), while 76% and 117% higher than OMW treated and control plants (*p* < 0.05), respectively.

## Anthocyanin metabolism

Table 4 illustrates the shifts in anthocyanin accumulation and their biosynthetic intermediaries, as well as two main enzymes involved in their biosynthesis pathway when the plants treated with AMF and OMW under oxidative stress caused by Sb contamination. Interestingly, there was no statistical difference (*p* > 0.05) in the anthocyanin content between stressed and non-stressed plants. Similarly, the content of phenylalanine, naringenin, and cinnamic acid in stressed control plants remained unchanged (*p* > 0.05) compared to non-stress conditions, however the activity of enzymes involved in their metabolisms significantly (*p* < 0.05) was improved by 39% in PAL and 36% in CHS in the stressed plants (Table 4).

**Table 4:** The effect of arbuscular mycorrhizal fungus (AMF) and olive mill waste (OMW) on some phenolic acid (mg g^−1^ DW), flavonoid compound (mg g^−1^ DW), phenylalanine amino acid (mg g^−1^ protein), and the activity of phenylalanine amnio lyase (PAL) enzyme (nkatal mg^−1^ protein) and chalcone synthase (CHS) (nmol mg^−1^ protein min^−1^) in oat plants

	Non-stress			Antimony stress			
	Control	OMW	AMF	Control	OMW	AMF	OMW + AMF
Anthocyanin	13.36 ± 1.04 c	13.14 ± 1.00 c	13.55 ± 0.95 c	13.15 ± 0.85 c	18.67 ± 1.66 ab	17.07 ± 1.45 b	21.41 ± 1.40 a
Phenylalanine	0.77 ± 0.09 b	0.74 ± 0.07 b	0.83 ± 0.10 b	0.97 ± 0.06 b	1.46 ± 0.11 a	1.42 ± 0.16 a	1.65 ± 0.11 a
Cinnamic acid	5.38 ± 0.37 b	5.29 ± 0.36 b	4.91 ± 0.44 b	5.69 ± 0.29 b	6.64 ± 0.36 ab	6.40 ± 0.37 ab	7.86 ± 0.47 a
Naringenin	0.30 ± 0.02 b	0.32 ± 0.02 b	0.35 ± 0.03 b	0.42 ± 0.03 b	0.40 ± 0.03 b	0.67 ± 0.05 a	0.62 ± 0.05 a
PAL	40.71 ± 3.15 c	42.57 ± 3.87 c	42.73 ± 3.29 c	56.40 ± 3.11 b	62.19 ± 5.88 ab	65.02 ± 4.38 ab	71.24 ± 3.38 a
CHS	4.21 ± 0.24 c	4.07 ± 0.20 c	6.15 ± 0.53 ab	5.85 ± 0.42 b	5.32 ± 0.45 b	7.33 ± 0.53 a	7.31 ± 0.56 a

The means in each parameter with similar small letter(s) are not significantly different at 5% probability level (Tukey's HSD test)

Nevertheless, anthocyanin and phenylalanine content in stressed plants were significantly (*p* < 0.05) increased by OMW (+ 42% and + 50%, respectively), AMF (+ 30% and + 46%, respectively), and OMW-AMF (+ 63% and + 69%, respectively) treatments compared to control. The content of individual phenolic acid (cinnamic acid) and PAL activity in the stressed plants reached the highest value in response to OMW + AMF treatment, which was 38% and + 26% (*p* < 0.05) higher than in stressed control plants, respectively. A significant increment (*p* < 0.05) in the content of individual flavonoid (naringenin) and CHS activity in the stressed plants was found in AMF (+ 59% and + 25%, respectively) and OMW + AMF (+ 47% and + 25%, respectively) treatments as compared to control (Table 4).

## Discussion

The present study attempted to deal with some unanswered questions regarding the impacts of soil heavy metals contamination on plants, one of which is the high levels of Sb in soil. Therefore, the responses of oat plants to Sb contamination were studied, especially to reveal whether the application of bio-inoculation with AMF and supplementation with OMW could alleviate the adverse effects of soil contamination through the modifications of some of the plant's biochemical and metabolite features.

The results evidently showed a lower accumulation of plant biomass under Sb contamination. The main explanation for such a reduction in biomass production can be related to a decline in photosynthesis rate and chlorophyll *a* content, as shown in Fig. 2. This finding is consistent with those of Zhou et al. [41], Duan et al. [42] and Khamis et al. [5], who reported unfavourable plant growth under high levels of soil Sb (1000–2000 mg kg^−1^ of soil) because of inhibition effects of Sb on photosynthetic pathways. In this regard, it has been reported that the Sb levels in plant cells can be affected by its high level in soil, and consequently decline photosynthetic efficiency by affecting the sulfhydryl group of chloroplast proteins and disrupting the structures and functions of chloroplasts [7, 43]. It has also been reported that the higher levels of chlorophyll content in stressed plants can protect the chloroplast structure and function from disruption and degradation [9]. Hence, it can be inferred that that the higher chlorophyll pigments in plants treated with OMW-AMF can be a reason for the higher photosynthesis rate and therefore biomass accumulation under contamination conditions compared to contaminated control plants.

Moreover, the fresh and dry weights significantly increased when exposed to the combined treatment of OMW and AMF in the contaminated soil, while the improvement was not found in the AMF-treated plants. These results need to be interpreted with caution since prior studies on rice and red clover plants noted that plants inoculation with AMF resulted in lower biomass production and photosynthetic efficiency when exposed to heavy metals compared to non-inoculated plants [44, 45]. They attributed this exacerbated negative effect of AMF to its symbiotic effects in higher absorption of heavy metals by plants from the soil through mycelia of AMF [46]. This is mainly can occur by extending the contact area of plant roots with Sb in soil [45, 47]. Soil acidifying in response to AMF exudes and transforming Sb from the carbonate minerals to soluble forms [48, 49], and increasing the soil electronegativity which directly impacts the speciation form of the metalloid Sb in soil [50].

Nevertheless, we did not detect significant effect of AMF treatment on biomass production, indicating that the reaction to AMF in the presence of heavy metals may depend on the plant species or even the type of variety [2, 51]. This symbiotic effect can be also affected by environmental growth factors, in particular soil pH [4]. Moreover, in line with the results of Xi et al. [4] and Khamis et al. [5], AMF inoculation contributes to the stabilization or increment of stress-related metabolic enzymes and the photosynthetic function at the protein level towards the incurred Sb stress. This can mean that despite the possible increase of Sb accumulation) in AMF-treated plants (not investigated in the present research, the AMF treatment can also be effective in reducing metal toxicity incurred by host plants [4].

More accumulation of H_2_O_2_ and MDA in plants grown under contamination can depict increased levels of ROS and lipid peroxidation. This finding confirms the reports of earlier studies, in which oxidative stress was found in Sb-stressed plants, mainly because the level of such oxidative stress may surpass the capacity of the antioxidant enzymatic pool [5, 52]. Moreover, an obvious decline in the accumulation of these oxidative damage markers in plants was observed in response to AMF inoculation and OMW treatment in the contaminated soil (Fig. 3). This result corroborates the results of a great deal of the previous research, in which the favourable effects of AMF and OMW treatments in declining the concentration of oxidative markers in stressed plants have been reported [5, 7, 19]. It seems possible that these results are due to the higher detoxification of ROS in AMF- and OMW-treated plants under stress, in which the antioxidant enzymes and molecules were more activated than those in stressed control plants.

The detoxification of overproduced ROS in plants has been reported to be attributed to the activation of the antioxidant defence strategies in response to OMW and AMF treatments [5, 19]. Similar reports revealed higher levels of direct ROS-detoxifying enzymes (POX, SOD, and CAT) and antioxidant enzymes and metabolites involved in the ASC/GSH (ASC, GSH, APX, GR, MDHAR, DHAR, and GPX) can ameliorate the damaging effects of oxidative stress caused by heavy metals contamination [8, 9, 19]. In this regard, the response of ASC and GSH in both OMW- and AMF-treated plants under Sb contamination in the present research, as the non-enzymatic component of the ASC-GSH pathway, coordinated with the activation of involved enzymes, especially APX, which catalyze the reduction of H_2_O_2_ into H_2_O [53]. Likewise, activating the ASC-GSH pathway in OMW- and AMF-treated plants was supposed a preliminary process in ROS detoxifying and preserving plant cells from oxidative damage [7, 54].

The high values of total antioxidant capacity, total flavonoids, total polyphenols and vitamins (tocopherols) in plats treated with OMW and AMF, are consistent with earlier findings, in which the higher levels of antioxidant metabolites content in the treated plants have been reported as an adaptation strategy against different environmental stress [9, 55, 56], in particular by acting as protective agents in preserving the photosynthetic apparatus [9]. In addition, the observed boost in the content of antioxidant metabolites in treated plants could be related to the higher accumulation of phenylalanine, as shown in Table 4, because of its function and role o as a precursor of numerous metabolites in plant defence systems against environmental stress such as flavonoids, polyphenols, and anthocyanins [57]. Moreover, the responses of phenylalanine and anthocyanins in this study confirm the shifts in the activity of the phenylalanine ammonia-lyase (PAL) enzyme, especially in OMW-AMF treatment (Table 4), which is responsible for catalyzing the deamination of phenylalanine as the foremost and rate-limiting stage of the phenylpropanoid pathway at the regulation point between primary and secondary metabolites [58]. Similarly, Xi et al. [4] reported that AMF can maintain the proteins involved in phenylalanine metabolism at a high level under Sb stress and modulate the adverse effects of stress on plant cells.

It is also clear that AMF could increase the activity of chalcone synthase (CHS) in the plants grown in the contaminated soil. The high level of CHS activity may explain the relative boost in both total flavonoids and naringenin content in AMF-treated plants since this enzyme acts as a central hub for the enzymes involved in the flavonoid pathway [59]. On the other hand, the individual application of OMW was not enough stimulus to enhance the activity of PAL and CHS enzymes, the stimulating effect that was observed in the combined treatment of OMW-AMF under stress conditions. The non-significant shift in naringenin content in OMW-treated plants, as an outcome of the flavonoid pathway (Table 4), can suggest the lack of non-competitively inhibition by this pathway in raising the enzyme activity [60] since it is proven that increased accumulation of naringenin in the cytosol can restrict CHS activity to evade toxic levels in the cells [61]. These results described the regulatory operation of such detoxifying metabolites in the present study, especially in AMF-treated plants, as one of the foremost protection systems under soil contamination.

## Conclusion

In the present study, a high level of soil Sb (1500 mg kg-1 of soil) revealed a detrimental impact on the oat plants through a considerable decline in biomass production and photosynthesis rate along with a rise in the levels of oxidative damage markers. In contrast, the combined treatment of arbuscular mycorrhizal fungus (AMF) and olive mill waste (OMW) significantly improved the biomass accumulation and photosynthesis capacity and induced the enzymatic and non-enzymatic antioxidants of treated oat plants grown under Sb contamination. Such positive effects were found in the individual application of these treatments to a lesser extent. Therefore, the findings suggest that the use of AMF in conjunction with OMW supplementation holds promise for improving plant growth and enhancing tolerance to oxidative stress induced by soil Sb contamination in agricultural settings.

## Data Availability

The data that support the findings of this study are available on request from the corresponding author.
